# Evaluation of the Efficiency of the Newly Developed Needle in Emergency Room: A Single-Center Observational Study

**DOI:** 10.2478/jccm-2024-0025

**Published:** 2024-07-31

**Authors:** Yuki Kishihara, Hideto Yasuda, Masahiro Kashiura, Takatoshi Oishi, Yutaro Shinzato, Takashi Moriya

**Affiliations:** Jichi Ika University Saitama Medical Center, Saitama-shi, Saitama Japan

**Keywords:** catheters, critical illness, emergency service, needles, quality improvement

## Abstract

**Aim of the study:**

Peripheral intravascular catheter (PIVC) insertion is frequently performed in the emergency room (ER) and many failures of initial PIVC insertion occur. To reduce the failures, new needles were developed. This study aimed to investigate whether the use of the newly developed needle reduced the failure of initial PIVC insertion in the ER compared with the use of the existing needle.

**Material and methods:**

This single-centre, prospective observational study was conducted in Japan between April 1, 2022, and February 2, 2023. We included consecutive patients who visited our hospital by ambulance as a secondary emergency on a weekday during the day shift (from 8:00 AM to 5:00 PM). The practitioners for PIVC insertion and assessors were independent. The primary and secondary outcomes were the failure of initial PIVC insertion and number of procedures, respectively. We defined the difficulty of titrating, leakage, and hematoma within 30 s after insertion as failures. To evaluate the association between the outcomes and the use of newly developed needles, we performed multivariate logistic regression and multiple regression analyses by adjusting for covariates.

**Results:**

In total, 522 patients without missing data were analysed, and 81 (15.5%) patients showed failure of initial PIVC insertion. The median number of procedures (interquartile range) was 1 (1–1). Multivariate logistic regression analysis revealed no significant association between the use of newly developed PIVCs and the failure of initial PIVC insertion (odds ratio, 0.79; 95% confidence interval, [0.48–1.31]; p = 0.36). Moreover, multiple regression analysis revealed no significant association between the use of newly developed PIVCs and the number of procedures (regression coefficient, −0.0042; 95% confidence interval, [−0.065–0.056]; p = 0.89).

**Conclusions:**

Our study did not show a difference between the two types of needles with respect to the failure of initial PIVC insertion and the number of procedures.

## Introduction

Peripheral intravascular catheter (PIVC) insertion is considered the most basic medical procedure and is performed in almost all cases, especially in the emergency department (ED) [1]. Complications of PIVC insertion include hematoma, superficial venous thrombosis, and skin inflammation and necrosis associated with drug leakage [2,3]. PIVC insertion is difficult in the ED, and failure of PIVC insertion occurs in 24–50% of cases [4,5,6]. Moreover, failure of initial PIVC insertion in the ED is associated with increased adverse events [7]. Thus, many failures of initial PIVC insertion occur in the ED, which may lead to several complications that may have a significant impact on patient management in the ED [1,3].

Previous studies have reported various risk factors for failure of PIVC insertion, which can be broadly classified into the following three categories: factors related to the practitioner, factors related to patients, and factors related to PIVCs [6,8]. These risk factors include the skill of the practitioner, visibility of veins, palpability of veins, mobility of veins, patients’ movements in violation of orders, and the gauge of catheters [6,8]. Recently, new needles were developed, and a previous study on non-human subjects using a vinyl chloride tube reported that the success insertion rate of new needles was 100%, while that of existing needles was 40% [9]. However, this study did not include human participants. When used on patients in clinical practice, it is expected that factors related to the patients such as visibility of veins, palpability of veins, mobility of veins, and patients’ movements in violation of orders will reduce the success rate of PIVC insertion. Therefore, studies on human participants using new needles in clinical practice are necessary.

If the use of the newly developed needle reduces the rate of failure of PIVC insertion in the ED, decreased failure rates and number of insertions may lead to decreased complications, which will be beneficial to patient care. Therefore, our study aimed to investigate whether the use of newly developed needles reduces the failure of initial PIVC insertion compared with the use of existing needles in the ED.

## Materials and Methods

### Study Design

We conducted a single-center, prospective observational study in Japan between April 1, 2022, and February 2, 2023. This study was pre-registered at UMIN-CTR under the Japanese Clinical Trial Registry (registration number: UMIN000045539) and was approved by the ethics committee of Jichi Medical University Saitama Medical Center (approval number: S22–030). As no interventions that deviated from clinical practice were performed in this study, and the structure of the newly developed needle and the existing needle differ only in the angle of the bevel. It was judged that the likelihood of new or specific complications arising from the use of the newly developed needle is extremely low. Therefore, the typical requirement for informed consent was waived. Nevertheless, we provided an opt-out procedure on the website of the Department of Emergency Medicine of the Jichi Medical University Saitama Medical Center.

This study was conducted according to the guidelines specified in the Strengthening the Reporting of Observational studies in Epidemiology (STROBE) statement as well as in accordance with the principles of the Declaration of Helsinki and its later amendments (e-Table 1 in Supplemental File 1) [10].

### Patients

The current study included consecutive patients who visited the Jichi Medical University Saitama Medical Center by ambulance as a secondary emergency on a weekday during the day shift (from 8:00 AM to 5:00 PM), with a minimum of two cases and no upper limit on the number of cases. These criteria were used to avoid selection bias. The exclusion criteria were as follows: 1) age < 18 years, 2) patients with cardiac arrest, 3) maintenance dialysis, 4) PIVC inserted by the first-grade resident, 5) difficulty in data collection, and 6) PIVC not inserted. Difficulty in data collection occurred in cases where PIVCs were inserted before data collection preparations were completed. These criteria were established to avoid bias due to factors related to the patient’s vessels or physician’s skills in inserting PIVCs.

### Data Collection

The following data were collected: age, sex, height, weight, body mass index (BMI), Charlson Comorbidity Index, presence of hypertension, presence of dyslipidaemia, Sequential Organ Failure Assessment score, insertion site (hand, forearm, elbow, upper arm, dorsal foot, and lower leg), visibility of veins, palpability of veins, mobility of veins, medical staff inserting the catheter (residents, physicians excluding residents, individuals with < 5 years of nursing experience, and individuals with ≥ 6 years of nursing experience), types of needles (newly developed and existing needles), catheter gauge (22G and 20G), echographic measurements of the major axis of veins, the ratio of the major axis of vessels to the diameter of PIVCs (major axis of vessels/diameter of PIVCs), patients’ movements in violation of orders, results of initial insertion, and the number of procedures performed until the success of insertion. We hypothesized that there would be a difference in the skill of inserting PIVCs between residents and grades above residents; thus, we classified physicians into residents and physicians excluding residents. In addition, based on information from the nurse performing the PIVC insertion in the Department of Emergency Medicine of Jichi Medical University Saitama Medical Center, the PIVC insertion technique was established in approximately the fifth year of nursing. Thus, we classified nurses into those with < 5 years and ≥ 6 years of nursing experience. Echographic measurements of the major axis of vessels were performed after applying a tourniquet because the diameter of the vessel after applying a tourniquet is thought to be related to the success or failure of PIVC insertion.

### Exposure

When patients were transported by ambulance as a secondary emergency to the Jichi Medical University Saitama Medical Center, the physician in charge of the current study reviewed the inclusion criteria. The included patients were allocated to the following two groups according to the type of needle (newly developed and existing needles): Surshield SurfloII with the thin-tipped short bevel needle named 3D-Shin^®^ (TERUMO CORPORATION, Tokyo, Japan) and Surshield SurfloII^®^ (TERUMO CORPORATION, Tokyo, Japan) [9]. The bevel angle of the new needle has been sharply angled from 30° of the existing needles to 20° to reduce resistance during cannulation and prevent deviation from vein (Table 1) [9]. The practitioner for PIVC insertion was selected at the discretion of the clinical site and separately from the physician in charge of the study. The selection of the types of needles (newly developed and existing needles) and catheter gauge (22G and 20G) was conducted by the practitioner, and no one else interfered with this selection. The practitioner performed PIVC insertion according to common insertion techniques. The physician in charge of the current study observed the insertion process and outcomes and collected data individually; however, the physician was not involved in the selection and insertion process. Therefore, data collection was unmasked, and the outcome assessment was unblinded.

**Table 1.** Structure of newly developed and existing needles

**Catheter gauge size**	**Types of needles (newly developed and existing needles)**	**Geometric design of the needle**	**Bevel length, mm**	**Bevel angle, degrees**
20G	Surshield SurfloII with the thin-tipped short bevel needle named 3D-Shin (newly developed needle)	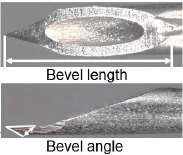	2.2	22
	Surshield SurfloII (existing needle)	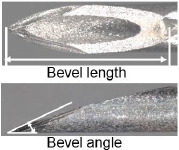	2.2	28
22G	Surshield SurfloII with the thin-tipped short bevel needle named 3D-Shin (newly developed needle)	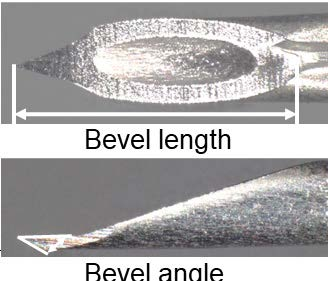	1.9	22
	Surshield SurfloII (existing needle)	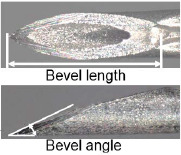	1.9	26

### Outcomes

The primary outcome was the failure of the initial PIVC insertion. We defined the difficulty of titrating, leakage, and hematoma within 30 s after insertion as failures. The secondary outcome was the number of procedures performed until the success of PIVC insertion. Regarding the number of procedures, data were collected only when the practitioner did not change.

### Sample Size

A previous study in humans reported that the failure rate of the first PIVC insertion was 24% [5]. Another study on newly developed needles using vinyl chloride tubes reported that the use of this needle reduced the failure rate by 60% as an absolute difference compared with the existing needles [9]. Given the 24% failure rate of the first insertion in humans in a previous study, a 60% reduction as an absolute difference in failure rate was considered invalid [5]. Therefore, considering its clinical importance, we conservatively estimated a 10% reduction as the absolute difference in the failure rate.

We calculated the sample size using two different methods and adopted a larger sample size. For the first method, we calculated the sample size by setting the statistical power and the α error. Given a 1:1 allocation, we determined that the enrolment of 522 patients would provide 80% power at a 2-sided α error level of 0.05 to detect statistically significant differences between the use of two needles. For the second method, we calculated the sample size based on the number of covariates included in the logistic regression analysis. We used eight covariates in the logistic regression analysis. For the logistic regression analysis, failure of initial PIVC insertion was required ten times the number of intended covariates, which was calculated to be 80 times. In the current study, the rate of failure of initial PIVC insertion was estimated to be 24% for the existing needle group and 14% for the newly developed needles group [5,9]. Assuming an average failure rate of 19% for the entire group, the sample size was calculated to be 421. Therefore, after comparing the results of these two calculations, the sample size was determined to be 522.

### Statistical Analyses

Continuous variables are described using medians and interquartile ranges, and categorical variables are described using absolute counts and percentages. Continuous variables were analyzed using the Mann–Whitney U test, and categorical variables were analyzed using Fisher’s exact test. We did not use imputation and analyzed using only complete cases.

To evaluate the association between the primary outcome and the use of newly developed needles and between the secondary outcome and the use of newly developed needles, we performed multivariate logistic regression and multiple regression analyses, respectively. Moreover, we extracted the following covariates based on previous studies for both analyses: visibility of veins, palpability of veins, mobility of veins, medical staff inserting the catheter (residents, physicians excluding residents, individuals with < 5 years of nursing experience, and individuals with ≥ 6 years of nursing experience), the ratio of the major axis of vessels to the diameter of PIVCs, and patients’ movements in violation of orders [6,7]. In addition, for the primary outcome, we created interaction terms by combining the types of needles and other explanatory factors and performed the logistic regression analysis. We performed stratified analysis as an interaction analysis for the interaction terms that were significant because of the logistic regression analysis. We assessed multicollinearity using the variance inflation factor (VIF). If the VIF was > 10, we considered multicollinearity and removed from the analysis any factor for which multicollinearity was observed. The effect estimates were described using odds ratios (ORs) and 95% confidence intervals (CIs), and a two-sided p-value of < 0.05 was considered the threshold for statistical significance.

All statistical analyses were performed using EZR version 1.38 (Saitama Medical Center, Jichi Medical University, Saitama, Japan), a graphical user interface for R (The R Foundation for Statistical Computing, Vienna, Austria), and the R statistical software (version 3.5.2; The R Project for Statistical Computing, Vienna, Austria) [11].

## Results

### Patients’ Characteristics

**Fig. 1.**
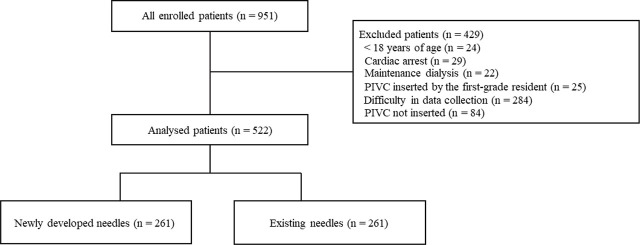
**Flowchart depicting the screening and enrolment process within this study.** PIVC, peripheral intravascular catheter.

Of the total of 951 enrolled patients, 522 patients were analyzed (Figure 1). The reasons for exclusion were as follows: 24 patients were aged < 18 years, 29 patients had cardiac arrest, 22 patients underwent maintenance dialysis, 25 patients were inserted PIVCs by the first-grade resident, data collection of 284 patients was difficult, and 84 patients did not undergo PIVC insertion.

The patient characteristics are shown in Table 2. Overall, the median age (interquartile range) was 73 (57–82) years, and 297 patients (56.9%) were male.

**Table 2.** Patient characteristics during emergency room arrival

**Variables**	**Overall (n = 522)**	**Newly developed needles (n = 261)**	**Exiting needles (n = 261)**	**p-value**
Age, median (IQR), years	73 (57–82)	73 (56–82)	73 (57–81)	0.94
Male sex (n, %)	297 (56.9)	142 (54.4)	155 (59.4)	0.29
Body height, median (IQR), cm	162 (155–168)	161 (155–168)	162 (155–169)	0.49
Body weight, median (IQR), kg	57 (50–67)	57 (50–66)	57 (50–68)	0.54
BMI, median (IQR), kg/m^2^	22.0 (19.5–24.2)	21.8 (19.1–24.3)	22.0 (19.5–24.2)	0.66
Charlson Comorbidity Index, median (IQR)	1 (0–2)	1 (0–2)	1 (0–2)	0.32
SOFA score, median (IQR)	0 (0–1)	0 (0–1.8)	0 (0–1)	0.93
Hypertension (n, %)	209 (40.0)	103 (39.5)	106 (40.6)	0.86
Dyslipidemia (n, %)	85 (16.3)	36 (13.8)	49 (18.8)	0.16

Insertion site (n, %)				
Hand	13 (2.5)	10 (3.8)	3 (1.1)	0.05
Forearm	385 (73.8)	185 (70.9)	200 (76.6)	0.44
Elbow	113 (21.7)	58 (22.2)	55 (21.1)	0.78
Upper arm	10 (1.9)	7 (2.7)	3 (1.1)	0.21
Lower leg	1 (0.2)	1 (0.4)	0 (0)	-^†^
Visibility of veins (n, %)	462 (88.5)	230 (88.1)	232 (88.9)	0.89
Palpability of veins (n %)	490 (93.9)	243 (93.1)	247 (94.6)	0.59
Mobility of veins (n, %)	249 (47.7)	126 (48.3)	123 (47.1)	0.86

Inserted by (n, %)				
Residents	5 (1.0)	3 (1.1)	2 (0.8)	0.65
Physicians excluding residents	37 (7.1)	19 (7.3)	18 (6.9)	0.87
Individuals with < 5 years of nursing experience	82 (15.7)	42 (16.1)	40 (15.3)	0.83
Individuals with ≥ 6 years of nursing experience	398 (76.3)	197 (75.7)	201 (77.0)	0.84
Catheter gauge (n, %)				
20G	330 (63.2)	161 (61.7)	169 (64.8)	0.53
22G	192 (36.8)	100 (38.3)	92 (35.2)	0.56
Major axis of veins, median (IQR), mm	3.0 (2.4–4.0)	3.0 (2.2–4.0)	3.2 (2.6–4.0)	0.02
Major axis of vessels/diameter of PIVCs, median (IQR)	3.1 (2.3–3.8)	3.0 (2.3–3.7)	3.2 (2.6–3.9)	0.04
Movements of patients (n, %)	27 (5.2)	13 (5.0)	14 (5.4)	1.0
Failure of initial insertion (n, %)	81 (15.5)	39 (14.9)	42 (16.1)	0.81
Number of procedures performed until the success of insertion^††^, median (IQR)	1 (1–1)	1 (1–1)	1 (1–1)	0.86

Abbreviations: BMI, body mass index; IQR, interquartile range; PIVC, peripheral intravascular catheter; SOFA, sequential organ failure assessment;

† This value could not be calculated;

†† This value was calculated only for cases in which the initial insertion failed.

This value could not be measured in 24 patients because of a change of practitioner.

The median BMI was 22.0 (19.5–24.2) kg/m^2^. Around 209 (40.0%) had hypertension, 85 (16.3%) had dyslipidaemia, and 385 (73.8%) had a PIVC inserted in the forearm. In total, 462 (88.5%) veins were visible, 490 (93.9%) were palpable, and 249 (47.7%) were movable. Moreover, in 398 (76.3%) patients, insertion was performed by an individual with ≥ 6 years of nursing experience, 330 (63.2%) needles were 20G, the median major axis of veins was 3.0 (2.4–4.0), and the median ratio of the major axis of vessels to the diameter of PIVCs was 3.1 (2.3–3.8). Overall, 81 (15.5%) patients had the failure of initial insertion, and the median number of procedures was 1 (1–1) times. There were no missing data (Table 2).

### Association of Needle Type with Failure of Initial PIVC Insertion or Number of Procedures

There was no significant association between needle type and failure of initial PIVC insertion in the multivariate logistic regression analysis using existing needles as a reference (OR, 0.79; 95% CI, [0.48–1.31]; p = 0.36) (Table 3). Other results of the logistic regression analysis for covariates are described in e-Table 2 in Supplemental File 2. Moreover, there was no significant association between needle type and the number of procedures in the multivariate multiple regression analysis using existing needles as a reference (regression coefficient, −0.0042; 95% CI, [−0.065–0.056]; p = 0.89) (Table 4). All VIFs were < 10, and there was no multicollinearity. Other results of the multiple regression analysis for covariates are described in e-Table 3 in Supplemental File 2.

**Table 3.** Result of logistic regression analysis for newly developed needles and failure of initial PIVC insertion

**Variables**	**Univariate analysis OR (95% CI)**	**p-value**	**Multivariable analysis OR (95% CI)**	**p-value**
Types of needles				
Existing needle	ref	-	ref	-
Newly developed needle	0.92 (0.57–1.47)	0.72	0.79 (0.48–1.31)	0.36

Abbreviations: CI, confidence interval; OR, odds ratio; PIVC, peripheral intravascular catheter.

**Table 4.** Result of multiple regression analysis for newly developed needles and the number of procedures^†^

**Variables**	**Univariate analysis Regression coefficient (95% CI)**	**p-value**	**Multivariable analysis Regression coefficient (95% CI)**	**p-value**
Types of needles				
Existing needle	ref	-	ref	-
Newly developed needle	0.00021 [(−0.059)–(0.063)]	0.95	−0.0042 [(−0.065)–(0.056)]	0.89

Abbreviations: CI, confidence interval; PIVC, peripheral intravascular catheter;

† This value could not be measured in 24 patients because of a change of practitioner.

### Interaction Analysis

We performed the logistic regression analysis by creating interaction terms by combining the types of needles and other explanatory factors. There was a statistically significant difference in insertion by physicians excluding residents, with individuals with ≥ 6 years of nursing experience as a reference. In subgroup analysis, the rates of failure of initial PIVC insertion for newly developed and existing needles for physicians excluding residents were 38.9% and 5.3%, respectively (e-Table 4 in Supplemental File 2).

## Discussion

This study analyzed 522 patients, and after multivariate logistic regression analysis and multiple regression analysis, there was no statistically significant association between the needle type and failure of initial PIVC insertion and between the needle type and the number of procedures, respectively.

We did not find the efficacy of the newly developed needles in the current study; however, this may be a matter of the population at risk. While a previous study reported 24% failure of initial PIVC insertion, the rate of failure of initial PIVC insertion in our study was much low, at approximately 15% [5]. Easy cases for insertion have factors such as palpable or visible veins, and the veins of approximately 90% of the patients included in the current study were palpable or visible; this percentage is considered high. Therefore, the patients included in the current study represented a population in which PIVC insertion was easy, and the failure rate was low. Difficult intravenous access (DIVA) is a global concept. Interventions for DIVA are the focus of the future worldwide; for example, ultrasound-guided insertion has been effective for DIVA [12,13]. In previous studies, various factors were thought to affect patients with DIVA: 1) no palpable or visible veins, 2) previous history of difficult venous catheterisation, 3) patient age < 4 years, and 4) tissue health (e.g. renal failure, diabetes, edema, and cachexia) [12,13]. With regard to the first factor, in our study, the veins of approximately 90% of the included patients were palpable or visible, and PIVC insertion was easy. Moreover, regarding the fourth factor, the patients included in our study were older adults and had skin that was not highly elastic. A previous study reported that PIVC insertion into highly elastic tissue is difficult because of needle flexion [14]. Moreover, previous studies have reported that the skin of older adults is less elastic than that of younger individuals owing to the decrease in collagen fibers, and insertion resistance is expected to decrease as elasticity decreases [9,15]. Therefore, the older patients included in our study had reduced skin elasticity, and the needle did not flex; thus, PIVC insertion may not have been difficult. In contrast, to reduce resistance during insertion and facilitate PIVC insertion, the bevel angle of the newly developed needles is 20° [9]. Comparing the newly developed and existing needles when inserting a vinyl chloride tube, the penetration forces at the needle and catheter tips of the newly developed needles were significantly lower than those of the existing needles, and the success rate of the initial PIVC insertion was improved [9]. The newly developed needles may be more effective than the existing needles, particularly for patients with DIVA with large vascular and skin elasticity. Patients whose vessels were palpable or visible and older patients included in our study were unlikely to benefit from the ease of insertion of the newly developed needles.

We did not find a statistically significant association between the use of newly developed needles and the failure of initial PIVC insertion in multivariate logistic regression analysis. However, given the advantages of the structure of newly developed needles, they may be more effective than the existing needles, and patients with DIVA are likely to benefit from the ease of insertion, particularly patients whose vessels are not palpable or visible. If studies were conducted on patients with DIVA, the results may differ from those of our study.

This study had several limitations. First, a significant difference between the newly developed and existing needles was not detected owing to the low power of this study. While a previous study reported 24% failure of initial PIVC insertion, the rate of failure of initial PIVC insertion in our study was much low, at approximately 15% [5]. Furthermore, the absolute risk reduction associated with the use of newly developed needles is calculated to be 29 patients per 1,000 (2.9%), converting the adjusted OR in our study to a risk ratio [16]. Given a 1:1 allocation, it was determined that the enrollment of 4,800 patients would provide 80% power at a 2-sided α error level of 0.05 to detect statistically significant differences between the two needles. However, the power was calculated to be 12% for the 522 patients in the current study. Therefore, a significant difference between the newly developed and existing needles was not detected because of the insufficient number of patients. Nevertheless, in the results of the subgroup analysis as an interaction analysis, there was a statistically significant difference in insertion by physicians excluding residents, with individuals with ≥ 6 years of nursing experience as a reference (e-Table 4 in Supplemental File 2). This significant difference may have been detected as an accidental error because of the insufficient sample size, and the interaction of the person performing the insertion may no longer be applicable if a sufficient number of patients were included. Second, the difference between the two needles in our study may have been detected if different subjective outcomes were measured. The bevel angle of the newly developed needles was 20°, and the penetration forces at the needle and catheter tips for the newly developed needles were low [9]. A previous study indicated that a decrease in skin resistance during insertion may reduce pain, and it is thought that the number of pain spots to be stimulated decreases because a low resistance reduces the extent of skin deformation during PIVC insertion [17]. The newly developed needles may be less painful than the existing needles because of their low penetration forces and resistance during PIVC insertion [9,17]. Therefore, differences between the newly developed and existing needles could have been detected if studies had been conducted considering different outcomes, such as pain scoring. Finally, external validity may be low because there were only a few patients with DIVA in our study. In a previous study that evaluated the risk of DIVA on a three-level scale of low, medium, and high, approximately 30% of the included patients were judged to be at medium and high risk [18]. The items used to classify risk into three levels in the previous study were palpable and visible veins. However, in our study, the veins of approximately 10% of the included patients were not palpable or visible, and this percentage is considered low compared with that in a previous study [18]. Moreover, BMI is thought to affect DIVA [13]. The median BMI in our study was 22.0 kg/m^2^. Given that the mean BMI in developed countries is approximately 25–30 kg/m^2^, the patients included in our study would be considered thin compared with those in other countries [19]. Therefore, the results of our study may not be applicable to patients with DIVA whose veins are not palpable or visible and whose BMI is high.

As a future perspective, a multi-center study including a larger number of patients might allow us to detect statistically significant differences between the newly developed and existing needles in terms of the failure of initial PIVC insertion that were not identified in the current study. Particularly, in the interaction analysis of the current study, the failure of initial PIVC insertion by physicians excluding residents were less frequent with the newly developed needle; however, the small sample size made multivariate logistic analysis difficult. Therefore, conducting a study with a larger number of patients might enable detection of statistically significant differences through multivariate logistic analysis. Conversely, given the ease of insertion based on the advantages of the structure of newly developed needles, the newly developed needles may also be effective in a low-addressability center with less experienced and less highly trained personnel. And as previously mentioned, the newly developed needles may be effective in cases of DIVA. Therefore, conducting a study specifically focused on low-addressability centers or cases with DIVA might still detect statistically significant differences between the newly developed and existing needles in terms of the failure of initial PIVC insertion.

## Conclusions

Our study did not show a difference between the newly developed and existing needles with respect to the failure of the initial PIVC insertion or the number of procedures.

## References

[1] Kagel EM, Rayan GM (2004). Intravenous catheter complications in the hand and forearm. J Trauma.

[2] Gregg SC, Murthi SB, Sisley AC, Stein DM, Scalea TM (2010). Ultrasound-guided peripheral intravenous access in the intensive care unit. J Crit Care.

[3] Millington SJ, Hendin A, Shiloh AL, Koenig S (2020). Better With Ultrasound: Peripheral Intravenous Catheter Insertion. Chest.

[4] Helm RE, Klausner JD, Klemperer JD, lint LM, Huang E (2015). Accepted but unacceptable: peripheral IV catheter failure. J Infus Nurs.

[5] van Loon FHJ, Buise MP, Claassen JJF, Dierick-van Daele ATM, Bouwman ARA (2018). Comparison of ultrasound guidance with palpation and direct visualisation for peripheral vein cannulation in adult patients: a systematic review and meta-analysis. Br J Anaesth.

[6] Lapostolle F, Catineau J, Garrigue B, Monmarteau V, Houssaye T, Vecci I (2007). Prospective evaluation of peripheral venous access difficulty in emergency care. Intensive Care Med.

[7] Kishihara Y, Yasuda H, Kashiura M, Moriya T, Shinzato Y, Kotani Y (2023). Impact of the failure of initial insertion of a peripheral intravascular catheter on the development of adverse events in patients admitted to the intensive care unit from the emergency room: A post hoc analysis of the AMOR-VENUS study. Acute Med Surg.

[8] Jacobson AF, Winslow EH (2005). Variables influencing intravenous catheter insertion difficulty and failure: an analysis of 339 intravenous catheter insertions. Heart Lung.

[9] Tanabe H, Kawasaki M, Ueda T, Yokota T, Zushi Y, Murayama R (2020). A short bevel needle with a very thin tip improves vein puncture performance of peripheral intravenous catheters: An experimental study. J Vasc Access.

[10] von Elm E, Altman DG, Egger M, Pocock SJ, Gøtzsche PC, Vandenbroucke JP (2014). The Strengthening the Reporting of Observational Studies in Epidemiology (STROBE) Statement: Guidelines for reporting observational studies. Int J Surg.

[11] Kanda Y (2013). Investigation of the freely available easy-to-use software ‘EZR’ for medical statistics. Bone Marrow Transplant.

[12] Poulsen E, Aagaard R, Bisgaard J, Sørensen HT, Juhl-Olsen P (2023). The effects of ultrasound guidance on first-attempt success for difficult peripheral intravenous catheterization: a systematic review and meta-analysis. Eur J Emerg Med.

[13] Paterson RS, Schults JA, Slaughter E, Cooke M, Ullman A, Kleidon TM (2022). Review article: Peripheral intravenous catheter insertion in adult patients with difficult intravenous access: A systematic review of assessment instruments, clinical practice guidelines and escalation pathways. Emerg Med Australas.

[14] Abolhassani N, Patel R, Moallem M (2007). Needle insertion into soft tissue: a survey. Med Eng Phys.

[15] Lee JH, Park J, Shin DW (2022). The Molecular Mechanism of Polyphenols with Anti-Aging Activity in Aged Human Dermal Fibroblasts. Molecules.

[16] Higgins JPT, Thomas J, Chandler J, Cumpston M, Li T, Page MJ (2019). Cochrane Handbook for Systematic Reviews of Interventions.

[17] Tamai S, Yasutaka H, Fujisaki K, Ono T (2015). Study of the quantification technique of the needle puncture resistance. The collection of papers by The Japan Society for Precision Engineering.

[18] Loon FHJV, Puijn LAPM, Houterman S, Bouwman ARA (2016). Development of the A-DIVA Scale: A Clinical Predictive Scale to Identify Difficult Intravenous Access in Adult Patients Based on Clinical Observations. Medicine (Baltimore).

[19] World Health Organization THE GLOBAL HEALTH OBSERVATORY. Explore a world of health data.

